# Effect of Resilience on Health-Related Quality of Life during the COVID-19 Pandemic: A Cross-Sectional Study

**DOI:** 10.3390/ijerph182111394

**Published:** 2021-10-29

**Authors:** Monira I. Aldhahi, Shahnaz Akil, Uzma Zaidi, Eman Mortada, Salwa Awad, Nisreen Al Awaji

**Affiliations:** 1 Department of Rehabilitation Sciences, College of Health and Rehabilitation Sciences, Princess Nourah Bint Abdulrahman University, Riyadh 84428, Saudi Arabia; SASAhmed@pnu.edu.sa; 2 Department of Laboratory Medicine, Clinical Physiology, Karolinska Institutet, SE-14186 Stockholm, Sweden; Shahnaz_161@hotmail.com; 3 Department of Health Sciences, College of Health and Rehabilitation Sciences, Princess Nourah Bint Abdulrahman University, Riyadh 84428, Saudi Arabia; uazaidi@pnu.edu.sa (U.Z.); EMMortada@pnu.edu.sa (E.M.); 4 Community, Environmental, Occupational Medicine Department, Faculty of Medicine, Zagazig University, Zagazig 44519, Egypt; 5 Department of Communication Sciences, College of Health and Rehabilitation Sciences, Princess Nourah Bint Abdulrahman University, Riyadh 84428, Saudi Arabia; Nnalawaji@pnu.edu.sa

**Keywords:** resilience, quality of life, pandemic, adults, Saudi Arabia, gender

## Abstract

The unprecedented outbreak of coronavirus disease 2019 (COVID-19) has caused a huge global health and economic crisis. The aim of the study was to examine the extent to which the resilience of a person is associated with the quality of life (QoL) of adults amongst Saudi Arabia. A cross-sectional study was conducted among a sample of adults in Saudi Arabia. A total of 385 adults voluntarily participated in and completed the survey. The quality of life was measured using the “World Health Organization QoL”. The “Connor-Davidson Resilience Scale” instrument was also used to assess resilience during the COVID-19 pandemic. Amongst the 385 participants, 179 (46%) showed a good QoL, and 205 (54%) reported a relatively poor QoL. The resilience was found to be significantly associated with QoL. The study further revealed that gender-based differences were dominant in the QoL; the men respondents reported a significantly higher QoL in all the domains in comparison to the women respondents. The gender, income, and psychological health and interaction effect of resilience and age explained 40% of the variance in the total score of QoL. In reference to the predictors of the physical health domain of QoL, resilience, gender, and psychological health were significantly associated with the physical health domain of the QoL (R^2^ = 0.26, *p* = 0.001). It was also noted that gender was not associated with the social relationships and environmental domains of QoL (*p* > 0.05). Findings showed a statistically significant association between the score of QoL and resilience, age, gender, income, and psychological health. These findings highlight the significant contribution of gender-based differences, psychological health, and resilience on the domains of QoL.

## 1. Introduction

Human civilization has experienced several pandemics, which have triggered changes in the society and affected different aspects of people’s well-being [1]. The most recent pandemic announced by the World Health Organization (WHO) [2] involves the highly contagious Coronavirus Disease 2019 (COVID-19) caused by the newly discovered severe acute respiratory syndrome coronavirus 2 [3]. To reduce the spread of COVID-19 and enhance public health, the WHO and United Nations have encouraged countries to introduce different types of precautionary measures, including strict hygiene routines, social distancing, and psychological health support [4]. As of 5 August 2020, the continuously increasing number of global infections and deaths reached about 14 million and 470,000, respectively [4]. The Kingdom of Saudi Arabia (KSA) was among the first countries to initiate an immediate implementation of precautionary measures; namely, lockdown, curfews, travel restrictions, prohibition of religious gatherings, and cancellations of ceremonies [5,6]. The preventive measures and restrictions imposed by the country are seen as effective means for the containment of the disease, although on the other hand, it triggered stress and anxiety amongst certain groups of individuals in the population [6]. In addition, the applied restrictions had a negative influence on people’s physical health, their psychological, emotional and social well-being as well as their quality of life (QoL) [7].

**Figure 1:**
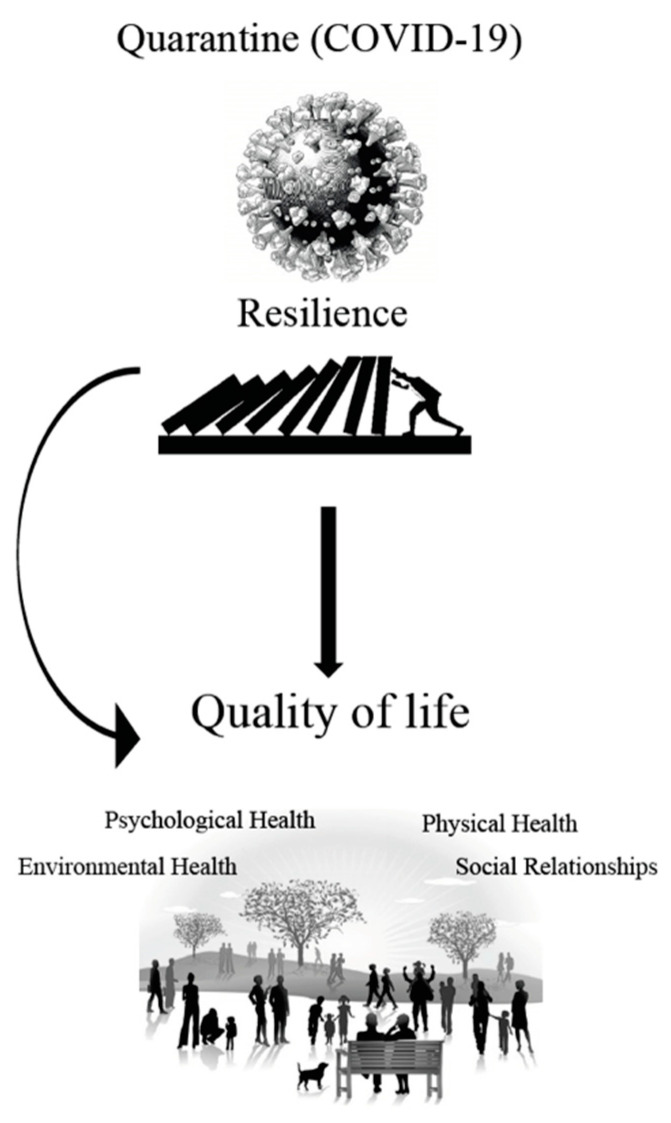
Shows a conceptual framework that depicts the hypothetical impacts of personal resilience, in reference to the aspects of quality of life during the COVID-19 pandemic.

An individual’s well-being is a combination of physical, psychological, and social health [8]. Therefore, the multidimensional nature of the concept of QoL includes an individual’s perception of several aspects of his or her well-being that goes beyond physical health [9] and makes it a significant variable to assess in the presence of global emergencies, such as the COVID-19 pandemic. One potent factor that may affect people’s QoL during this time is an individual’s ability to cope with the new living situation generated by this novel pandemic (Figure 1). A person’s ability to cope with and adapt to new life circumstances, challenges, or crises is known as resilience [10]. The concept of resilience has been studied for decades and has been found to be a protective mechanism that is triggered in the presence of adversity or a challenge [11].

Several studies have been carried out to investigate the mediating role of resilience in the relationship between negative factors and different aspects of an individual’s well-being [12,13]. For instance, indicators of psychological illness like depression and anxiety are associated with poor resilience [14], whereas the absence of psychological illness is known to be correlated to a higher level of resilience [15]. Furthermore, the relationship between resilience and QoL in a stressful environments or a fatal disease had previously been proven [10,12]. Published studies have shown that, resilience impacts QoL in certain populations with some diseases such as cancer [16], multiple sclerosis [10] and Parkinson’s disease [17] amongst others. However, data regarding to the relationship between resilience and the different aspects of QoL in the COVID-19 era are not yet conclusive (Figure 1).

One important aspect of QoL is the socioeconomic status (income, occupation, and education) of individuals, which is an important variable that should be taken into consideration when assessing the resilience during a specific shocking event in life, such as the presence of infectious diseases [18,19]. For instance, households in China with lower income, education and social security do not have access to proper healthcare and tend to show lower resilience during COVID-19, as compared to households with higher income and a higher level of education [19]. Furthermore, the presence of resilience characteristics in individuals may also be influenced by other factors such as gender. A previous study showed that women expressed a higher level of fear and anxiety, compared to men, as a reaction to specific stressors [20]. From a physiological perspective, the gray-matter volume of both genders in reaction to a specific event was compared in one study with the conclusion that men showed lower gray matter volume due to their higher resilience [21]. Thus, women and men tend to have different levels of resilience, as they have shown different psychological responses to various stressful events including COVID-19 [22,23]. The differences in resilience previously found between the genders [22] will create room for further studies to explore this association.

Hence, the main aim of this study was twofold. The first was to gain a better understanding of the characteristic of the health-related quality of life and personal resilience, with the main focus being on gender-based differences in the adult population. Second, was to examine the extent with which the resilience of a person is associated to the QoL of adults amongst the KSA, during the COVID-19 pandemic. The findings of this study may further highlight the importance of resilience in buffering against a decline in the health-related QoL in the context of COVID-19. A clear understanding of the factors that influence various aspects of people’s well-being is indeed essential, in order to boost public health during any pandemic.

## 2. Materials and Methods

### 2.1. Study Design and Population

This was a cross-sectional survey study, which was carried out between the month of May and August 2020 during the COVID-19 pandemic in the KSA. The study invitation text and link were distributed via different methods (Twitter and email) to adults aged between 18 years and above living in the KSA. A sample size calculation was employed using an unbiased sample estimator approach, which was calculated using the appropriate statistical package; namely, OpenEpi [24,25]. A sample of at least 340 adults was estimated based on the assumption of % frequency of 28.2% of respondents reported the lowest scores of QoL in previous study [26], with margin of error at 5%, z-score equals 1.96, confidence index level set to 95% and a design effect of 3 as we followed non-probability sampling. Participant’s consent was obtained at the beginning of the survey, and the study inclusion criteria were stated in the survey, include being aged older than 18 years and living in Saudi Arabia and having not been diagnosed with any physical or intellectual disabilities. Non-probability sampling method was implemented during the recruitment process, where 503 individuals responded to the questionnaire, and only 385 individuals managed to complete the entire questionnaire; of which 57% were women and the rest 43% were men. A total of 118 respondents were excluded, as they declined to participate, as well as respondents who did not fulfill the inclusion criteria (i.e., were below 18 years of age or reported a physical disability as these factors may confound the findings and required to be addressed differently using different questionnaires).

### 2.2. Study Instruments/Measures

The method used in the research was mainly quantitative in nature, a questionnaire was administered online using Survey Monkey. The survey link was distributed across the region of kingdom of Saudi Arabia and circulated via social media platforms. Individuals who lack access to social platform had been recruited via telephone communication. All respondents were encouraged to send the survey link to their relatives or friends. The survey comprised of a section related to the respondents’ sociodemographic and general health information and two self-reported questionnaires to assess the participants’ perception of their QoL and resilience during the COVID-19 pandemic. The following are the details of the questionnaires.

#### 2.2.1. Sociodemographic and Health Characteristics

A total of nine questions pertaining to sociodemographic and general health information were asked. The participants were asked to report their age, nationality, gender, educational qualifications (high school, bachelor’s degree, postgraduate studies), marital status (married and unmarried) and the perceived economic status (low input, middle input, high input) were included in the survey. The questions which were related to the respondents’ general psychological health (absence of psychological illness, presence of psychological illness), presence or absence of disabilities, and physical health status (absence of health problems, presence of health problems) were also added.

#### 2.2.2. Health-Related Quality of Life

The QoL was examined using the Arabic-translated and standardized version of the “*World Health Organization Quality of Life Questionnaire (WHOQOL-BREF*)” [27]. This instrument contains 26 items which had been validated previously [27]. The questionnaire was clarified further by rephrasing a few items to specify how they are related to the perception of QoL during the COVID-19 pandemic. Thus, a total of 26 items were used in this study, which yielded scores in five domains: 2 items for general QoL and health status, 7 items for physical health, 6 items for psychological, 3 items for social relations, and 8 items regarding the environment health. All the items ranked using a five-point Likert scale on each item with a higher score indicating a better QoL. The Arabic translation of the WHOQOL-BREF reported to have a high significance and showed an excellent internal consistency (α = 0.95) reliability. For the purpose of analyzing the data, the 3 negatively framed items, we reversed to positively framed ones. Then each domain score was calculated; all scores were multiplied by 4 to be comparable with scores derived from the WHOQOL-100. Finally, by using the transformation table provided in the used manual, we converted the raw scores to transformed ones [27]. The transformed scores were used for the statistical analyses of the four domains of the WHOQOL-BREF, whereas each raw domain score was converted to scores ranging from 4–20 to be consistent with the WHOQOL-100 instrument, according to the transformation method outlined in the WHOQOL-BREF scoring instructions [27]. In our present study, WHOQOL-BREF demonstrated a good internal consistency, with a Cronbach’s Alpha coefficient (α) of 0.86.

#### 2.2.3. Resilience

The resilience of the participants was assessed using the “*Connor-Davidson Resilience Scale (CD-RISC)*” [28]. The questionnaire included 10 items in both Arabic and English [29]. Permission to use the questionnaire was obtained from Dr. Jonathan Davidson. The items on the CD-RISC-10 were subjectively used to assess how the participants felt during the COVID-19 lockdown using a five-point Likert-type scale [30]. The CD-RISC involves 10 items that describe personal flexibility, optimism and self-efficacy under stress, ability to regulate emotion, and cognitive focus under stress which showed an excellent psychometric properties [30]. The total possible scores ranging from 0–40, with a higher score indicating higher personal resilience and lower scores indicating less resilience. In our present study, *CD-RISC* demonstrated a good internal consistency, with a Cronbach’s Alpha coefficient (α) of 0.85.

### 2.3. Compliance with Ethical Standards

The study was reviewed and granted ethical approval by the Institutional Review Board, at Princess Nourah bint Abdulrahman University [IRB: 20-0191] in Riyadh, Saudi Arabia. A description of the study and the email address of the principal investigator were included at the beginning of the survey. A consent question was added to ensure approval from the respondents to take part in the study.

### 2.4. Statistical Analysis

The collected data were analyzed using the Statistical Package for the Social Sciences (SPSS), version 20 (IBM Corp., Chicago, IL, USA). The normality distribution of the dependent variables was measured using a Kolmogorov–Smirnov test. Descriptive statistics were run and expressed as the mean ± standard deviation (SD) for normally distributed variables, and as the median [interquartile range (IQR)] for non-normally distributed variables. The reliability of the total WHOQOL-BREF scale (26 items), its four domains, and the resilience scale were assessed using the Cronbach’s alpha coefficient; in which an alpha coefficient of 0.70 or greater was considered an acceptable internal consistency.

Consequently, Kruskal–Wallis and Mann–Whitney U tests were also conducted to compare the median scores, since the domains of the QoL were skewed. The median of the outcome variables (level of QoL) in the current study was considered a cutoff point to categorize either a poor QoL (median ≤ 66) or a good QoL (median > 66), as there was no previously agreed-upon cutoff point. In addition, individual’s resilience was stratified into two categories: low resilience with a median of 24 or less and a high resilience with a median greater than 24, based on the set cutoff point for the median resilience.

The Spearman’s rho correlation coefficient was also calculated to determine the level of relationship between the four domains of the WHOQOL-BREF and the resilience scale. To understand the association of resilience and other demographic variables on QOL, hierarchical linear regression models were carried out, in which the total QoL score was taken as the dependent variable. The resilience was included in the first model, and in the second model gender was added, whereas the third model factored in income while the last model combined income and psychological problems as a variable. Changes in the R2 were compared in the models to understand the variance as explained by the resilience in the QoL. A multiple regression analysis was then carried out to explain the contribution of each independent predictor, in the variance in the domains of QoL. The models included all variables that were significant in the bivariate analyses, and the significance level *p*-value of ≤0.05 was set for all the statistical analysis in the entire study.

## 3. Results

### 3.1. Participants Sociodemographic Characteristics

From a total of 385 respondents who completed the survey and provided electronic informed consent; 219 (57%) were women, and 166 (43%) were men. Table 1 shows the descriptive statistics of the participants. The findings revealed that most of the participants, roughly 291 (75.6%), reported no physical problems. Regarding the educational level, the collected data revealed that most of the participants, that is, 286 (74.3%) had a bachelor’s degree, whereas the rest (76, representing 19.7%) had a postgraduate degree. In addition, 230 (60%) were younger than 45 years old. A total of 300 participants (77.9%) reported having an average income, and 255 participants (66.2%) were married. The collected data further revealed that 355 (92.2%) of the participants had no psychological health problems.

### 3.2. Health-Related QoL

The median score of the QoL was 66, ranging from 31 to 78 with an IQR of 12. Meanwhile, the medians of the domains of the QoL were as follows: 14 for physical health with a range of 6–19, 14 for psychological health with a range of 7–19, 16 for social relationship with a range of 4–20 and 15 for environmental domain with a range of 5–20. Table 2 shows the differences in the total QoL, and its domains separately based on resilience level and health problems (either psychological or physical). A further analysis of the difference in the domains of QoL based on gender is reported in Table 3. The data showed that men’s total scores of QoL were significantly higher than women’s QoL score (*p* = 0.003). As reported in Table 3, there were significant differences in all domains of QoL between men and women.

In Table 4, a total of 54% of participants showed a poor QoL and 46% reported a relatively good QoL, with a QoL score above the median score of the pooled sample. Further comparison was carried out between the group of poor QoL and of good QoL is shown, which reveals that the categories of age, marital status, income level, and psychological health and resilience were significantly different (*p* < 0.05). Among the participants who claimed to have a good QoL, a minority, 5 (2.8%), reported having psychological health problems, while only 39 (21.9%) reported to be having physical health problems. In contrast, among the participants who reported a poor QoL, only 25 (12.1%) had psychological health problems and 55 (26.6%) had physical health problems. There was a significant main effect of the level of income on QoL levels and a lack of significant difference in the educational level groups and the self-identified physical health problem of the respondents (Table 4).

### 3.3. Resilience

The median score of the resilience was 24 (range: 6–40), with an IQR of 8. The resilience score significantly differed between groups with good and poor QoL. As indicated in Table 4, most of the participants with a good QoL reported high resilience scores. The median resilience score was significantly higher in the good QoL group than in the poor QoL group (27 (IQR: 7) vs. 22 (IQR: 6), respectively; *p* <0.001). Table 3 does not display any gender-based differences in resilience. The resilience score did not significantly differ between gender groups.

### 3.4. Correlations between QoL, Resilience and Sociodemographic Characteristics

All the correlations are presented in Table 5. The correlation analysis between resilience and different sociodemographic characteristics indicated that age, gender, marital status, income and psychological health problems were correlated significantly with the participants’ resilience (all *p* < 0.05).

### 3.5. Predictors of QoL and Resilience

In Table 6, significant factors from the bivariate analysis were entered into the model of multiple linear regression analyses. The analysis showed a lack of significant association between the educational level and all domains of QoL as dependent variables (all *p* > 0.05), while the age variable did not demonstrate statistically significant associations with the physical health domains of QoL (*p* = 0.13). The analyses further revealed that gender was not associated with the social relationship and environmental health domains of QoL (all *p* > 0.05). Table 7 shows the results of the hierarchical multiple linear regression analysis, which reveals that resilience was significantly associated with QoL (β = 0.53, R^2^ = 0.28, *p* < 0.001). Notably, when gender, income level, psychological problems, and Age × Resilience were added, the full model had a good fit to the data and explained 40% of the total variance in the QoL (F (5) = 50.29, *p* < 0.001), as shown in Table 7. The analysis showed a statistically significant interaction effect between age and resilience on the total score of QoL.

Table 8 displays the predictors of the four domains of QoL. It was shown that resilience predicted the four domains of QoL (physical, psychological, and environmental health and social relationships). In terms of the predictors of the physical health domain of QoL, only resilience, gender, and psychological health were significantly associated with the physical health domain of the QoL (R^2^ = 0.26, *p* < 0.001), as indicated in Table 8. Subsequent analyses revealed that only age, resilience, income, and psychological problems are significantly associated with the environmental and social relationships of QoL ((F = 39.39, R^2^ = 0.29); (F = 27.90, R^2^ = 0.23), all *p* < 0.001, respectively).

## 4. Discussion

The rapid and unprecedented spread of COVID-19 has had staggering effects on all sectors of the economy and has affected the lives of individuals worldwide. Because of the unforeseen circumstances, financial recessions, and lockdowns, psychological health professionals have warned about the effects of the pandemic upon physical and psychological health, social relationships, and environmental health [31]. In particular, adverse effects on psychological well-being are expected, along with high rates of stress, anxiety, and depression among the general population [32,33]. Under these circumstances, it is essential to explore factors that may reduce the negative impacts of the COVID-19 pandemic on overall QoL. Although the topic of QoL and resilience had been studied in the past [34], there is a dearth of literature regarding pandemics and predictors of QoL. The current study aimed to characterize QoL in the adult population and investigate the gender-based differences in QoL amid the COVID-19 pandemic. Further investigation was conducted to assess the association between resilience, sociodemographic factors, and QoL. Most respondents indicated that they had a poor QoL, and women reported lower scores of QoL. The results showed that resilience was a factor that influenced variance in QoL. These findings indicated that people with high resilience responded actively to the current situation and, in turn, demonstrated a good QoL. Moreover, certain sociodemographic variables, such as gender, income, and psychological health, were also significant predictors of QoL.

The current COVID-19 pandemic has raised concerns about health-related QoL. An individual’s perception of QoL can be affected by various situations. Most of the existing literature agrees that situations including disability, health-related issues, disease, or living with a person suffering from a terminal disease ultimately diminish scores in the overall perceptions of QoL. During recent lockdowns due to COVID-19, most daily activities were suspended or became limited in scope. This further strengthened the assumption that QoL might be severely impacted during the COVID-19 pandemic [34]. Upon further investigation of QoL during the COVID-19 pandemic, this study revealed that more than half of the participants (*n* = 207, 54%) reported a relatively poor QoL. The findings observed in this study mirror those of the previous study that reported a significant effect of the COVID-19 pandemic on various aspects of QoL in the KSA [35]. This trend is in line with cultural tendencies in the KSA; a recent study examining QoL among mothers of children with or without disabilities in the KSA showed similar results [36].

In addition, the effect of gender-based differences on QoL is a matter of escalating concern in the KSA. Indeed, the present study revealed that gender-based differences affected QoL; most men respondents reported a higher QoL than women. Additionally, the gender is one of the factors that predicted the total score of QoL in this study. Thus, the findings did not reveal any significant difference in levels of resilience between men and women. However, the findings of the current study do not support the previous work which demonstrated that compared to women, men were almost two times more likely to report lower scores of QoL [35].

The results revealed no difference in resilience factor between men and women participants. However, women respondents scored lower on QoL. This might be explained through the lens of cultural context, where women usually get married during their university level study and simultaneously carry the responsibilities of taking care of family and their career. Moreover, women have relatively fewer opportunities to engage in outdoor activities as compared to their men counterparts, which could influence their perception of QoL and well-being [37]. This implies that society’s existing gender differences can, to some extent, influence health [37]. Even though at governance level, many policies have been introduced to strengthen women’s QoL, full implementation is needed in order to make progressive change. During the pandemic, the preventive measures taken by the KSA were the same for both the genders. Another reason that cannot go unnoticed is that during the pandemic, women were burdened with more household responsibilities without any additional support. For professional women, this became more difficult to manage in combination with office duties that had to be performed from home and the needs of family members that had to be balanced. Ultimately, this situation can produce stress. In future studies, it will be interesting to study the correlation of QoL with other factors, especially in women. Moreover, a comparison between married women with children, married women without children, and single women could reveal the causes of low-level physical problems and their impact on QoL. However, this was not the main goal of the study; therefore, future research is recommended to explore these factors.

Resilience is a psychological coping mechanism that helps people actively respond to adversity [10,11,12]. Resilience is frequently studied across various disciplines and has been shown to buffer against detrimental disturbances or life events; it improves an individual’s ability to cope and bounce back from negative events [38,39]. From a theoretical perspective, resilience is considered a relevant issue with regard to the pandemic; it is a defensive measure that can buffer factors that mitigate well-being. In this study, it is reported that 83% of the participants who self-reported psychological health issues (*n* = 30) demonstrated a poor QoL. Positive psychological resources such as resilience may facilitate and promote the well-being and physical health of individuals [40,41]. The levels of resilience in the present study varied greatly, especially in individuals who reported a good vs. poor QoL, confirming the assumption that resilience is associated with QoL.

In parallel with a previous study [42], the current study found resilience to be high. It also revealed that being resilient can allow one to respond positively to a stressful situation, enabling the maintenance of psychological and physical well-being. This is also in agreement with findings highlighted in previous studies [43,44]. The results of the current study demonstrated that people with higher levels of resilience indicate a higher QoL and that differing resilience levels can explain variance in overall QoL levels. These findings support the idea that the resilience is a type of psychological capital that facilitates better psychological health [45]. The findings of the present study highlight the association of the psychological domain of QoL with resilience, which is in agreement with a previous study carried out among medical students which revealed that resilience correlated positively with QoL [46]. Prior studies have indicated an indirect relationship between resilience, depression, and loneliness [47,48]. Empirical findings in patients with heart failure and depression revealed that resilience increased psychological health and, to some extent, alleviated depressive symptoms [49]. However, some demographic factors can affect this relationship and should therefore be taken into consideration. Our study’s findings confirmed that some demographic characteristics (low income and poor psychological status) were also correlated with resilience. In contrast, Waugh and Koster stated that psychological problems do not necessarily imply failed resilience from a personal perspective [50]. However, it is still unclear whether poor psychological health implies low resilience or vice versa. Future prospective and follow-up studies are necessary in order to address this concern.

Other factors may influence QoL which is an area of great concern due to unprecedented dynamic lifestyle changes due to COVID-19. It is unknown whether the dynamic of life during the COVID-19 pandemic has contributed, to some extent, to the QoL levels. In Saudi Arabia, multisectoral approaches for COVID-19 containment have been implemented. Public health systems have made a massive effort to contain emerging COVID-19 through the use of surveillance systems and contact tracing as crucial elements to controlling the pandemic. The intersectoral collaboration between all areas of public health, payers, healthcare providers and non-health sectors (education, security, finance, industry, legislative, public works, habitat), and community coordinated their effort to promote good health and control the spread of the virus [5,51]. Concerted efforts from the public health system and healthcare providers were offered, and the requirement of those with minor symptoms to seek care and to quarantine themselves were critical requirements for effective infection control. Hospitals were equipped and subsidized testing was made available for any patients even with mild symptoms; the pocket costs were waived, which potentially helped in managing any further transmission [5,51]. The government implemented strategic preparedness and further implemented partial lockdowns and postponed visits to holy places and mosques in most KSA regions [5,52]. These shifts in lifestyle may have contributed to the reported finding regarding the total health-related QoL.

Upon further investigation, the present study revealed that income levels explain variability in the social, environmental, and psychological domains of QoL, while to some extent, gender differences can predict, to some extent, physical and psychological domains of QoL. The findings also suggested that other social factors and their complex interplay should be considered. In the present study, resilience was associated with all the examined domains of QoL. Resilience explained 28% of the variation in QoL, whereas gender, income, and psychological health accounted for 9%. Educational level did not demonstrate associations with the total QoL. This is in contrast to the findings of a previous study [53] that addressed health-related QoL in adults and its association with educational level; that study indicated that poorer perceived health status could be attributed to a lower educational level, which may vary by sex [53]. This incongruence between our results and previous findings may be due to differences in the constructed measures and cultural differences across studies.

There are additional significant intervening variables that could contribute to QoL. The degree of lockdown, varying severity of the pandemic in different geographical locations should be considered when interpreting the findings of the present study. Therefore, in the future, it is necessary to consider the contribution of personal factors (e.g., physical disability and psychological conditions, social support, and community integration). The findings of the present study suggested that it is beneficial to monitor various dimensions (e.g., gender and income level) with respect to resilience level in order to demonstrate the need to implement specific interventions.

This study revealed significant findings with regard to resilience and QoL; however, there are some limitations that need to be acknowledged. Non-probability sampling does not guarantee that the sample is representative of the KSA. Future research that uses a probability survey is recommended. The study only included individuals who were ≥18 years of age. Future research might explore the relationship between resilience and QoL in children and adolescents. Therefore, the findings of this study cannot be generalized due to the broad age range and the convenience sampling technique employed in the study Moreover, the cross-sectional design of the study has some limitations as it does not support the causal relationship between the exposure and outcome; thus, a longitudinal study design is required to capture the cause-and-effect relationship. Due to the sudden nature of the COVID-19 pandemic, it was not possible to perform a longitudinal study to investigate the impact of COVID-19 on QoL and explore the change in resilience that could be attributed to COVID-19. There are many other factors linked with QoL in addition to resilience, including well-being, coping styles, and community integration. In future studies, these factors should be investigated in relation to QoL. A larger sample size would also be beneficial in order to be able to generalize our results. The lack of information regarding the participants’ individual experience during COVID-19 pandemic (i.e., the extent to which they followed the quarantine rules, whether they went to work or not) prevents any analysis related to the influence of the participants’ experiences during the COVID-19 pandemic on their responses to the questionnaires. Another limitation is that many respondents were highly educated, which affects the generalizability of the study results. Lastly, this study was conducted to find out the effects of resilience on QoL during the pandemic in the general population. However, among the participants, no data were collected from any participant who had been diagnosed with COVID-19. In a future study, conducting a study on COVID-19 survivors should be considered in order to understand the impact of COVID-19 in more depth.

## 5. Conclusions

The present study characterized the QoL and examined the impact of resilience on QoL among adults in the KSA during the COVID-19 pandemic. It sheds light on factors affecting QoL during the COVID-19 pandemic, including resilience, age, gender, income, and psychological health. In summary, statistically significant results were found with respect to resilience and QoL. A total of 54% of participants showed a poor QoL and 46% reported a relatively good QoL, with a QoL score above the median score of the pooled sample. The median resilience score was significantly higher in the good QoL group than in the poor QoL group, although this score does not recognize the gender-based differences in resilience. The total QOL score did significantly differ between gender groups. Participants with high levels of resilience responded dynamically to the present situation, which to some extent, resulted in high QoL. There was a statistically significant association between the score of QoL and resilience, age, gender, income, and psychological health, which explained 40% of the variability in QoL. Factors such as age, gender, education, income, and psychological status were also correlated positively with resilience.

The results strongly show that resilience may act as a protective quality, reducing the negative impacts of adverse conditions that could decrease an individual’s perceived QoL. Concurrently, the findings of this study suggest the importance of considering an individual’s personal capability to adapt to tough situations, which can affect QoL. Hence, it is critical to recognize the groups most at risk of facing negative impacts of the pandemic on the QoL. The findings of this study suggest the need to design a wellness program that can enhance personal resilience to overcome the eminent impact of the pandemic on the well-being of communities. These findings highlight the significant contribution of gender-based differences resilience on health-related QoL, which may direct health care professionals in tailoring health promotion programs.

## Figures and Tables

**Table 1:** Participant characteristics (N = 385).

Variable	Responses	Frequency (%)
Age range (Years)	18–25	94 (24.4)
	26–35	68 (17.7)
	36–45	68 (17.7)
	46–55	71 (18.4)
	56–60	52 (13.5)
	>60	32 (8.3)
Educational level	High School	23 (6.0)
	Bachelor’s degree	286 (74.3)
	Postgraduate degree	76 (19.7)
Marital status	Married	255 (66.2)
	Unmarried	130 (33.8)
Income	Low	35 (9.1)
	Average	300 (77.9)
	High	50 (13.0)
Physical health problems	Yes	94 (24.4)
	No	291 (75.6)
Psychological problems	Yes	30 (7.8)
	No	355 (92.2)
Resilience (CD-RISC)	Low	163 (42.3)
	High	222 (57.7)

Abbreviation: CD-RISC, Connor-Davidson Resilience Scale.

**Table 2:** A comparison of the WHOQOL-BREF Scores according to the physical, psychological problems and resilience of the respondents and their level of resilience.

Characteristics	Responses	Total WHOQOL-BREF				
		DOM1 Mdn (IQR)	DOM2 Mdn (IQR)	DOM3 Mdn (IQR)	DOM4 Mdn (IQR)	Total Mdn (IQR)
Physical problems ^U^	Yes	14 (3)	14 (2)	14 (4)	15 (3)	65 (12)
	No	14 (3)	14 (2)	16 (4)	15 (3)	67 (12)
	*p* value	0.34	0.65	0.24	0.55	0.62
Psychological problems ^U^	Yes	12 (3)	12 (4)	12 (4.5)	13 (3.3)	54 (12.8)
	No	14 (3)	14 (2)	16 (4)	15 (3)	67 (11)
	*p* value	<0.001 *	<0.001 *	<0.001 *	<0.001 *	<0.001 *
Resilience (CD-RISC) ^U^	High	15 (3)	15 (2)	16 (4)	16 (3)	69 (10)
	Low	13 (4)	13 (3)	14 (4)	14 (2)	63 (13)
	*p* value	<0.001 *	<0.001 *	<0.001 *	<0.001 *	<0.001 *

* significant level *p* ≤ 0.05; ^U^ = Mann-Whitney U test; Mdn = Median; IQR = interquartile range; Abbreviations: DOM1, physical health; DOM2, psychological health; DOM3, social relationships; DOM4, environmental health; WHOQOL-BREF, World Health Organization Quality of Life-Brief; CD-RISC, Connor-Davidson Resilience Scale.

**Table 3:** A comparison of the WHOQOL-BREF domains and resilience scale based on the gender differences of the study respondents during the COVID 19 pandemic (N = 385).

Characteristics	Gender				*p* Value ^U^
	Men *n* = 166		Women *n* = 219		
	Mdn (IQR)	Mean Rank	Mdn (IQR)	Mean Rank	
QoL domains					
Physical Health	15 (2.2)	208.7	14 (3)	181.1	0.02 *
Psychological Health	14 (2)	215.1	14 (3)	176.2	0.001 *
Social Relationships	16 (2)	205.3	14 (4)	183.7	0.05 *
Environmental Health	15 (3)	210.8	14 (3.5)	179.5	0.006 *
WHOQOL-BREF total	67 (11)	212.4	65 (14)	178.3	0.003 *
Resilience score	25 (7.3)	200.9	24 (8)	186.9	0.22

* Significant level *p* ≤ 0.05; ^U^ = Mann-Whitney U test; Mdn = Median; IQR = interquartile range; Abbreviations: M, mean; SD, standard deviation; Mdn, Median; IQR, interquartile range; WHOQL-BREF, World Health Organization Quality of Life-Brief.

**Table 4:** A comparison of the personal characteristics of the respondents and their resilience based on their level of QoL (N = 385).

Characteristics		Level of QOL		TotalN (%)	X^2^ Test	*p* Value
		Poor *n* = 207	Good *n* = 178			
		N (%)	N (%)			
Age range (Years)	18–25	54 (26.1)	40 (22.5)	94 (24.4)	23.8	<0.001 *
	26–35	46 (22.2)	22 (12.4)	68 (17.7)		
	36–45	44 (21.3)	24 (13.5)	68 (17.7)		
	46–55	35 (16.9)	36 (20.2)	71 (18.4)		
	56–60	18 (8.7)	34 (19.1)	52 (13.5)		
	>60	10 (4.8)	22 (12.4)	32 (8.3)		
Educational level	High School	11 (5.3)	12 (6.7)	23 (6.0)	1.5	0.47
	Bachelor’s degree	159 (76.8)	127 (71.3)	286 (74.3)		
	Postgraduate degree	37 (17.9)	39 (21.9)	76 (19.7)		
Marital status	Married	126 (60.9)	129 (72.5)	255 (66.2)	5.8	0.02 *
	Unmarried	81 (39.1)	49 (27.5)	130 (33.8)		
Income	Low	26 (12.6)	9 (5.1)	35 (9.1)	24.6	<0.001 *
	Average	169 (81.6)	131 (73.6)	300 (77.9)		
	High	12 (5.8)	38 (21.3)	50 (13.0)		
Physical health problems	Yes	55 (26.6)	39 (21.9)	94 (24.4)	1.13	0.29
	No	152 (73.4)	139 (78.1)	291 (75.6)		
Psychological problems	Yes	25 (12.1)	5 (2.8)	30 (7.8)	11.4	0.001 *
	No	182 (87.9)	173 (97.2)	355 (92.2)		
Resilience score (CD-RISC)	Low	116 (56.0)	47 (26.4)	163 (42.3)	34.42	<0.001 *
	High	91 (44.0)	131 (73.6)	222 (57.7)		

* significant level *p* ≤ 0.05; data presented as frequency (N) and percentage (%); Abbreviations: QOL, quality of life; CD-RISC: Connor-Davidson Resilience Scale.

**Table 5:** A matrix of the Spearman correlation between the WHOQOL-BRE, personal characteristics, and resilience among the study respondents.

Variables	Correlation Coefficients								
	1	2	3	4	5	6	7	8	9
WHOQOL-BREF scale	1								
CD-RISC	0.51 **	1							
Age	0.24 **	0.22 **	1						
Gender	−0.15 **	−0.14 **	−0.06	1					
Marital status	−0.19 **	−0.13 **	0.01	−0.69 **	1				
Education	0.09	0.12 *	0.16 **	0.13 **	−0.07	1			
Income	0.30 **	0.34 **	0.16 **	0.24 **	−0.15 **	−0.21 **	1		
Health problems	0.04	0.03	0.03	−0.33 **	0.14 **	0.13 *	−0.04	1	
Psychological problems	0.26 **	0.21 **	0.17 **	0.11 *	−0.09	−0.08	0.02	0.09	1

* Correlation is significant at the 0.05 level. ** Correlation is significant at the 0.01 level. Abbreviations: WHOQOL-BREF, the World Health Organization Quality of Life-Brief; CD-RISC, the Connor-Davidson Resilience Scale.

**Table 6:** Simple linear regression analysis of the QoL and the four domains as dependent variables (N = 385).

Predictors	Physical Health		Psychological		Social Relationships		Environmental		Total QoL	
	B	95% CI	B	95% CI	B	95% CI	B	95% CI	B	95% CI
Age	0.13	−0.04–0.29	0.26	0.14–0.38 *	0.38	0.16–0.59 *	0.33	0.19–0.47 *	1.40	0.84–1.96 *
Resilience	0.18	0.15–0.22 *	0.14	0.11–0.17 *	0.24	0.19–0.30 *	0.16	0.13–0.20 *	0.82	0.69–0.95 *
Gender	−0.63	−1.12–−0.15 *	−0.69	−1.0–−0.29*	−0.75	−1.4–0.04	−0.64	−1.11–0.17	−3.02	−4.88–−1.16 *
Income	0.41	−0.05–0.88	0.88	0.46–1.30 *	1.64	0.90–2.37 *	1.75	1.28–2.23 *	6.05	4.15–7.95 *
Education	0.36	−1.33–0.86	0.44	0.03–0.85	0.34	−0.38–1.01	0.62	0.14–1.10	2.0	0.09–3.90
Psychological problems	2.39	1.51–3.26 *	2.23	1.52–2.95 *	2.40	1.11–3.69 *	1.88	1.02–2.74 *	9.96	6.63–13.30 *

* Significant level *p* ≤ 0.05. B: unstandardized beta “regression coefficient”; CI: Confidence interval.

**Table 7:** Hierarchical multiple linear regression analysis for the total WHOQOL-BERF score as dependent variable.

Model	Predictors	Coefficients ^a^						R^2^	R^2^_change_	VIF
		B	β	T	*p* Value	95% CI				
						Lower	Upper			
Model 1	Constant ^b^	45.3		26.9	<0.001 *	42.01	48.63	0.28	0.28	
	Resilience	0.82	0.53	12.21	<0.001 *	0.69	0.95			1.00
Model 2	Constant ^c^	49.5		23.04	<0.001 *	45.3	53.71	0.29	0.01	
	Resilience	0.81	0.52	12.15	<0.001 *	0.68	0.94			1.00
	Gender	−2.5	−0.13	−3.07	0.002 *	−4.06	−0.89			1.00
Model 3	Constant ^d^	41.8		14.12	<0.001 *	35.96	47.5	0.34	0.05	
	Resilience	0.8	0.49	11.67	<0.001 *	0.64	0.8			1.02
	Gender	−1.9	−0.10	−2.45	0.02 *	−3.46	−0.36			1.02
	Income	4.4	0.22	5.19	<0.001 *	2.75	6.1			1.04
Model 4	Constant ^e^	31.5		8.17	<0.001 *	23.92	39.1	0.37	0.03	
	Resilience	0.72	0.46	10.93	<0.001 *	0.59	0.85			1.06
	Gender	−1.7	−0.09	−2.13	0.03 *	−3.18	−0.13			1.03
	Income	4.25	0.21	5.08	<0.001 *	2.60	5.9			1.04
	Psychological problems	5.9	0.17	4.05	<0.001 *	3.02	8.71			1.05
Model 5	Constant ^f^	38.48		7.47	<0.001 *	23.92	39.1	0.40	0.03	
	Resilience	0.88	0.39	8.68	<0.001 *	0.59	0.85			1.31
	Gender	−1.7	−0.09	−2.02	0.04 *	−3.54	−0.05			1.49
	Income	5.70	0.20	4.81	<0.001 *	2.60	5.9			1.08
	Psychological problems	8.97	0.18	4.39	<0.001 *	3.02	8.71			1.05
	Age × Resilience	0.04	0.15	2.81	0.005 *	0.01	0.07			1.88

* Significant level *p* ≤ 0.05; B: unstandardized beta “regression coefficient”; β: standardized beta; VIP: Variance Inflation Facto. ^a^. Dependent Variable: WHOQOL-BREF. ^b^. Predictors: (Constant), resilience. ^c^. Predictors: (Constant), resilience, Gender. ^d^. Predictors: (Constant), resilience, Gender, Income. ^e^. Predictors: (Constant), resilience, Gender, Income, Psychological problems. ^f^. Predictors: (Constant), resilience, Gender, Income, Psychological problems, (Age × Resilience).

**Table 8:** Multiple linear regression models with the four domains of WHOQOL-BREF as dependent variables among study respondents (N = 385).

Dependent Variables	Predictors	Coefficients							R^2^
		B	SE	*β*	t	*p* Value	95% CI		
							Lower	Upper	
Physical Health	CONSTANT	7.45	0.92		8.04	<0.001 *	5.63	9.28	0.26
	Resilience	0.17	0.01	0.43	9.57	<0.001 *	0.14	0.21	
	Gender	−0.44	0.21	−0.09	−2.03	0.04 *	−0.86	−0.01	
	Psychological problems	1.54	0.41	0.17	3.78	<0.001 *	0.74	2.34	
Psychological	CONSTANT	7.07	0.89		7.90	<0.001 *	5.31	8.83	0.27
	Resilience	0.12	0.02	0.36	7.95	<0.001 *	0.09	0.15	
	Age	0.14	0.07	0.11	2.02	0.04 *	0.004	0.28	
	Gender	−0.19	0.22	−0.05	−.88	0.38	−0.63	0.24	
	Income	0.43	0.19	0.10	2.22	0.02 *	0.05	0.82	
	Psychological problems	1.51	0.34	0.20	4.47	<0.001 *	0.84	2.17	
Social Relationships	CONSTANT	4.34	1.34		3.25	0.001 *	1.72	6.97	0.23
	Resilience	0.22	0.03	0.38	8.27	<0.001 *	0.17	0.28	
	Age	0.24	0.10	0.11	2.37	0.01 *	0.04	0.44	
	Income	0.97	0.35	0.13	2.75	0.006 *	0.28	1.66	
	Psychological problems	1.09	0.61	0.08	1.82	0.07	−0.09	2.29	
Environmental	CONSTANT	6.33	0.86		7.38	0.000 *	4.64	8.01	0.29
	Resilience	0.14	0.02	0.36	8.01	0.000 *	0.11	0.17	
	Age	0.19	0.07	0.13	2.91	0.004 *	0.06	0.32	
	Income	1.29	0.23	0.26	5.72	0.000 *	0.85	1.74	
	Psychological problems	0.94	0.39	0.11	2.41	0.016 *	0.17	1.70	

* p ≤ 0.05 is significant; B: unstandardized beta “regression coefficient”; β: standardized beta, WHOQOL-BREF: World Health Organization Quality of Life-Brief.

## Data Availability

Data will be available on request.
